# Synthesis of Sb_2_S_3_ NRs@rGO Composite as High-Performance Anode Material for Sodium-Ion Batteries

**DOI:** 10.3390/ma14247521

**Published:** 2021-12-08

**Authors:** Hosung Hwang, Honggyu Seong, So Yi Lee, Joon Ha Moon, Sung Kuk Kim, Jin Bae Lee, Yoon Myung, Chan Woong Na, Jaewon Choi

**Affiliations:** 1 Department of Chemistry and Research Institute of Natural Science, Gyeongsang National University, Jinju 52828, Korea; hshwang3@gnu.ac.kr (H.H.); gu9188@gnu.ac.kr (H.S.); oi_sioy115@gnu.ac.kr (S.Y.L.); answns36@gnu.ac.kr (J.H.M.); sungkukkim@gnu.ac.kr (S.K.K.); 2 Korea Basic Science Institute, Daejeon 34133, Korea; jblee@kbsi.re.kr; 3 Dongnam Regional Division, Korea Institute of Industrial Technology, Busan 46744, Korea; myungyoon@kitech.re.kr

**Keywords:** Sb_2_S_3_ NRs@rGO, anode materials, reduced graphene oxide, sodium ion batteries

## Abstract

Sodium ion batteries (SIBs) have drawn interest as a lithium ion battery (LIB) alternative owing to their low price and low deposits. To commercialize SIBs similar to how LIBs already have been, it is necessary to develop improved anode materials that have high stability and capacity to operate over many and long cycles. This paper reports the development of homogeneous Sb_2_S_3_ nanorods (Sb_2_S_3_ NRs) on reduced graphene oxide (Sb_2_S_3_ NRs @rGO) as anode materials for SIBs. Based on this work, Sb_2_S_3_ NRs show a discharge capacity of 564.42 mAh/g at 100 mA/g current density after 100 cycles. In developing a composite with reduced graphene oxide, Sb_2_S_3_ NRs@rGO present better cycling performance with a discharge capacity of 769.05 mAh/g at the same condition. This achievement justifies the importance of developing Sb_2_S_3_ NRs and Sb_2_S_3_ NRs@rGO for SIBs.

## 1. Introduction

Until now, secondary batteries are widely used as ecofriendly energy storage. Specifically, the increasing electric car market has accelerated the development of secondary battery materials that have high capacity and stability to endure long-term electrochemical cycling [1,2,3,4,5]. To achieve these goals, it is essential to improve the anode’s storage capacity and thus, many possible materials have been tested [6]. Although lithium ion batteries (LIBs) meet the requirements for high energy storage devices with properties of high energy density and voltage, there are limitations in using lithium because of its high price and low earthly deposits [7,8]. As a substitute to LIBs, sodium ion batteries (SIBs) have been predicted as promising batteries because of their plentiful reserves and similar energy storage mechanism to LIBs [7]. However, the large diameter of the sodium atom leads to poor diffusion efficiency of Na^+^ and expands the anode material’s volume while intercalating [9]. Therefore, it is challenging to find a proper anode material that maintains good stability with the conditions described above [10]. Among these materials, antimony sulfide (Sb_2_S_3_) is one of the strongest anode candidates that has high gravimetric energy density and theoretical capacity (946 mA/g) [11]. However, there are downsides to Sb_2_S_3_ regarding its low conductivity and volume expansion during the sodiation/desodiation process when using Sb_2_S_3_ as energy storage [12]. To solve these problems, homogeneous-width Sb_2_S_3_ nanorods (Sb_2_S_3_ NRs) and graphene oxide (GO) were used to make Sb_2_S_3_ NRs@rGO anchored on reduced graphene oxide (rGO) [13,14,15,16]. Uniform sized Sb_2_S_3_ NRs could compensate volume variation to some degrees while intercalation of Na+ than various sized nanorods [17,18,19]. Reduced graphene oxide (rGO) layers not only have enough interstitial spots to receive Na^+^ but also have substantial conductivity making electron transfer easily. Moreover, the rigid structure of rGO buffer the stress of volume expansion on Sb_2_S_3_ NRs not to make dropwise of discharge capacity of the electrode over the long term. These traits of rGO invalidate the drawback of Sb_2_S_3_ and enable the intercalation reversibly with a large amount of Na^+^ raising capacity of the electrode [20]. 

Herein, this research reports the synthesis of homogeneous-width Sb_2_S_3_ NRs and Sb_2_S_3_ NRs@rGO. Through various electrochemical investigations, Sb_2_S_3_ NRs@rGO showed improved discharge capacity of 769.05 mAhg^−1^ at a current density of 100 mA/g after 100 cycles. Even at a higher current density of 500 mAg^−1^, excellent stability could be observed after 300 cycles, which was shown to be better than Sb_2_S_3_ NRs@rGO.

## 2. Materials and Methods

### 2.1. Synthesis of Sb_2_S_3_ NRs

First, 10 mL of oleylamine (OAm, from Sigma-Aldrich, St. Louis, MO, USA) was heated at 150 °C for an hour under vacuum conditions to remove impurities. After heating OAm, sulfur (S, 0.0412 g, from Sigma-Aldrich, St. Louis, MO, USA) and antimony chloride (SbCl_2_, 0.0661 g, 99%, from Sigma-Aldrich, St. Louis, MO, USA) were added to the well-dried OAm. Then the solution was heated to 230 °C over a period of 40 min with stirring and washed with 15 mL of methanol and hexane by centrifugation four times after cooling to room temperature. After that, dark gray Sb_2_S_3_ NRs powder was retrieved [21]. 

### 2.2. Synthesis of Graphene Oxide (GO) 

GO was prepared by the well-known Hummers method using graphite powder [20]. First, graphite (Super P, 2 g, from Timcal Ltd., Bodio, Switzerland) was added to a solution of sulfuric acid (H_2_SO_4_, 10 mL, from Samchun, Pyeongtaek, Korea), potassium persulfate (K_2_S_2_O_8_, 2 g, from Sigma-Aldrich, St. Louis, MO, USA), and phosphorus pentoxide (P_2_O_5_, 2 g, from Sigma-Aldrich, St. Louis, MO, USA) at 85 °C. After stirring the mixture for 5 h, it was cooled to room temperature and 500 mL of deionized (DI) water was added for then stirred for another 12 h. The mixture was washed with DI water under vacuum filtration and dried under vacuum at room temperature. The as-prepared products were put into a cold solution of sulfuric acid (H_2_SO_4_, 100 mL, from Samchun, Pyeongtaek, Korea) while cooling in an ice bath. Potassium manganate (KMnO_4_, 10 g, from Sigma-Aldrich, St. Louis, MO, USA) was added gradually to the resultant slurry during 1 h keeping in an ice bath. After removing an ice bath, the mixture was stirred for 24 h at room temperature. Then, 30% hydrogen peroxide (H_2_O_2_, 20 mL, from Samchun, Pyeongtaek, Korea) and DI water (500 mL) were mixed into the solution. After filtration of mixed solution, the resulting precipitate was rinsed with 10 wt % hydrochloric acid (HCl, 1 L, from Samchun, Pyeongtaek, Korea) solution in water followed by washing with DI water (1 L) to remove the acidic components.

### 2.3. Synthesis of Sb_2_S_3_ NRs@rGO

Prepared GO was dispersed in ethanol (EtOH, 15 mL, from Samchun, Pyeongtaek, Korea) and DI water (5 mL) using ultrasonication for 1 h. Sb_2_S_3_ NRs (10 mg) were also dispersed in ethanol (EtOH, 5 mL) the same way and the two solutions were together, followed by ultrasonication for 1 h. After that, hydrazine (N_2_H_4_, 0.2 mL, from Kanto Chemical, Tokyo, Japan) was added to the solution and placed in a 250 °C oven for over 3 h and sealed with electrical tape to make reduced GO (rGO). After the supernatant liquid was removed, the retrieved product was washed with ethanol three times and dried, finally generating black/gray Sb_2_S_3_ NRs@rGO powder.

### 2.4. Structural Refinement

A scanning electron microscope (SEM, JSM-7601F, JEOL, Tokyo, Japan) equipped with an energy dispersive spectrometer (EDS, Ultim Max, Oxford Instruments, Abingdon on Thames, UK) and transmission electron microscope (TEM, FEI RF30ST, Philips, Amsterdam, Netherlands) were used to observe the morphologies and structures of the sample. X-ray diffraction (XRD, D8 Advance A25, Bruker, Billerica, MA, USA) was used to characterize the composition of the samples at 40 kV and 40 mA with a 0.02 s/step. Raman spectra of the samples were obtained using a Raman instrument (Renishaw InVia, Renishaw, Wotton-under-Edge, UK) with a wavelength of 633 nm. X-ray photoelectron spectroscopy (XPS) was measured using a Thermo VG scientific Sigma Probe spectrometer (Sigma probe, Thermo VG scientific, East Grinstead, UK) with a monochromatic photon energy of 1486.6 eV (Al Kα).

### 2.5. Electrochemical Investigation

The half-coin cell (CR-2032) were assembled in a glove box filled with Ar to avoid oxygen and moisture (O_2_ < 1 ppm, H_2_O < 1 ppm) contact with the Na metal used as the counter electrode. The electrode was made by mixing Sb_2_S_3_ NRs (80 wt %) with polyvinylidene fluoride (PVDF) (10 wt %) binder and super P (10 wt %). A solution of 1 M NaPF_6_ in DEGDME is used as the electrolyte in SIBs. For comparison, Sb_2_S_3_ NRs were also made in the same way to assess the electrochemical performance. The test of galvanostatic charge-discharge was conducted in the voltage range of 0.01–3.0 V. Cyclic voltammetry (CV) measurements were carried out on a potentiostat (Zive SP1, Wonatech) at a voltage range of 0.01–3.0 V and a scanning rate of 0.05 mV/s. All electrochemical experiments above were performed at room temperature.

## 3. Results and Discussion

### 3.1. Morphology, Structure and Composition Analysis

**Figure 1:**
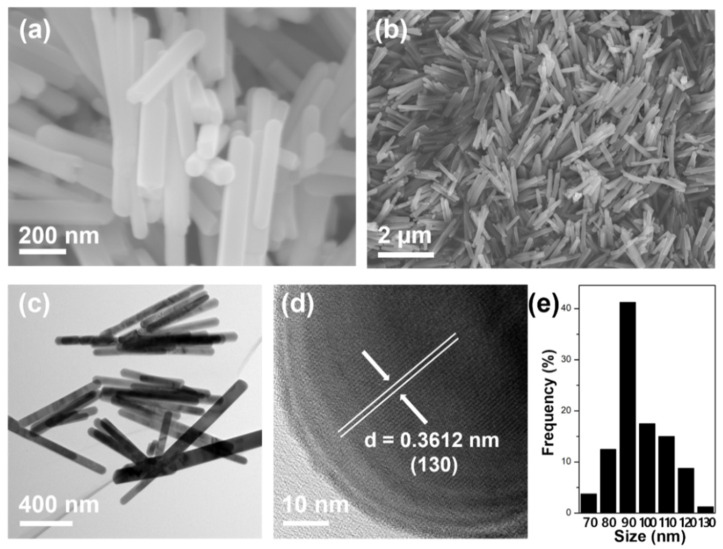
SEM Images of Sb_2_S_3_ NRs at magnification of (**a**) 80,000× and (**b**) 20,000×. TEM images of Sb_2_S_3_ NRs at magnification of (**c**) 12,000× and (**d**) 400,000×. (**e**) Size distribution of Sb_2_S_3_ NRs.

The shape of the Sb_2_S_3_ NRs was measured by SEM and TEM. As shown in Figure 1a,b, the Sb_2_S_3_ NRs synthesized by the hydrothermal method have smooth surfaces, showing good structural integrity. Figure 1c,d show TEM images of Sb_2_S_3_ NRs, which show a 0.36 nm lattice corresponding with the crystal plane of Sb_2_S_3_ (JCPDS No. 42-1393) [22]. These nanorods have a homogeneous-width average of 90 nm as determined via size distribution Figure 1e. 

**Figure 2:**
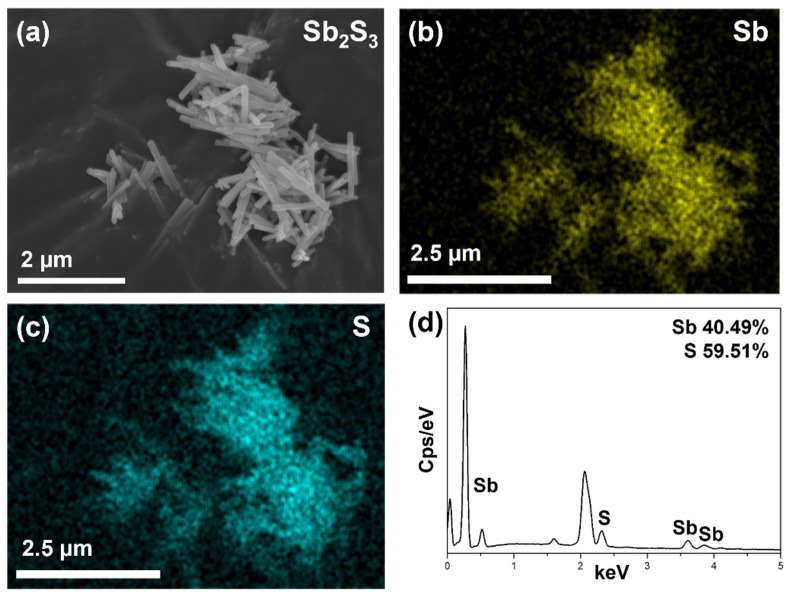
(**a**) SEM data of Sb_2_S_3_ NRs. Elemental mapping images of (**b**) Sb and (**c**) S. (**d**) EDS spectrum of Sb and S.

Mapping data of previous SEM images are shown in Figure 2. The SEM image of Sb_2_S_3_ NRs Figure 2a corresponds to the images of Sb Figure 2b and S Figure 2c, demonstrating homogeneity of the synthesized materials. As seen in the EDS data of Figure 2d and Table S1, 59.51 at% and 40.49 at% represent S and Sb, respectively, which proves the successful synthesis of Sb_2_S_3_ NRs (Table S1).

**Figure 3:**
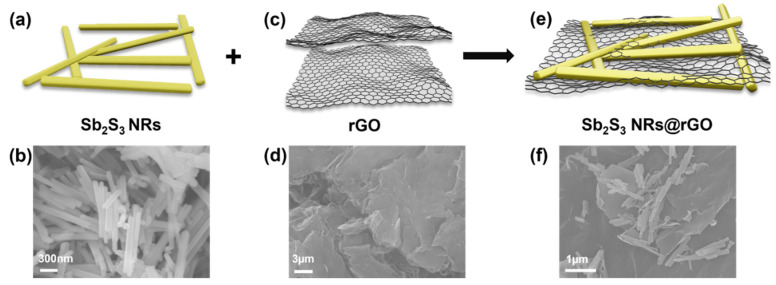
Schematic illustration of the composition with Sb_2_S_3_ NRs and reduced graphene oxide (rGO). (**a**,**b**) Illustration and SEM images of Sb_2_S_3_ NR. (**c**,**d**) Illustration and SEM images of reduced graphene oxide. (**e**,**f**) Illustration and SEM images of Sb_2_S_3_ NRs@rGO.

**Figure 4:**
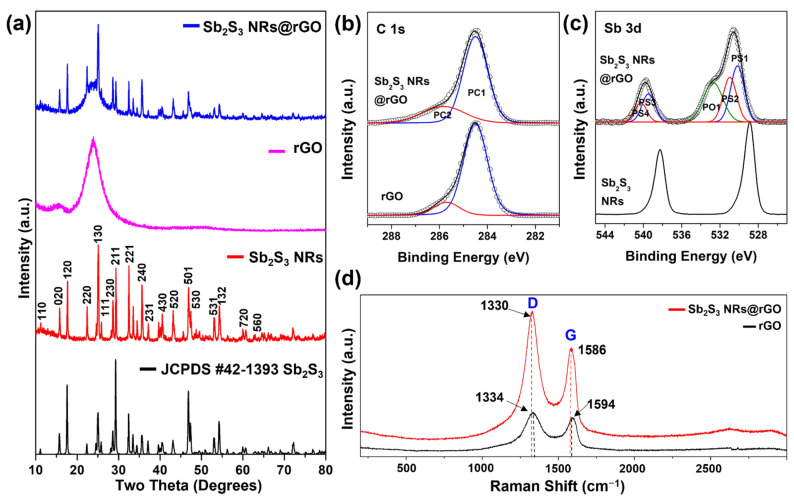
(**a**) XRD of Sb_2_S_3_ NRs, Sb_2_S_3_ NRs@rGO. (**b**) Fine-scanned C 1s of rGO and Sb_2_S_3_ NRs@rGO and (**c**) Sb 3d of Sb_2_S_3_ NRs and Sb_2_S_3_ NRs@rGO. (**d**) Raman spectrum of Sb_2_S_3_ NRs, Sb_2_S_3_ NRs@rGO.

The modified Hummers method to synthesize the Sb_2_S_3_ NRs@rGO composite is illustrated in Figure 3. Prepared Sb_2_S_3_ NRs Figure 3a,b are anchored to rGO Figure 3c,d, where the Sb_2_S_3_ NRs particles are covered with rGO layers Figure 3e,f. Figure 4a shows the X-ray diffraction (XRD) patterns of Sb_2_S_3_ NRs, rGO, and Sb_2_S_3_ NRs@rGO. The diffraction peaks of Sb_2_S_3_ NRs correspond to the diffraction pattern of Sb_2_S_3_ (JCPDS No. 42-1393) [22], and a comparison of each peak corresponds to either Sb_2_S_3_ NRs or rGO. The electronic surroundings of C and Sb in rGO, Sb_2_S_3_, and Sb_2_S_3_ NRs @rGO were compared by X-ray photoelectron spectroscopy (XPS) in Figure 4b,c, Figure S1, Tables S2 and S3. The C 1 s orbital peaks of rGO and Sb_2_S_3_ NRs @RGO were deconvoluted into the PC1 (C-C bonding) and the PC2 (C-O bonding) bands. The C 1s orbital peaks of rGO were observed at 284.5 eV, 285.7 eV, and Sb_2_S_3_ NRs @rGO were observed at 284.5 eV, 285.8 eV. Compared to rGO, the area of PC1 decreased from 86.7% to 75.1% while that of PC2 increased from 13.3% to 24.9%. The Sb 3d_5/2_ and Sb 3d_3/2_ orbital peaks of Sb_2_S_3_ NRs were observed 528.9 eV (Sb 3d_3/2_) and at 538.2 eV (Sb 3d_5/2_) respectively. Sb_2_S_3_ NRs@rGO was deconvoluted into five bands, a PS1 at 530.2 eV and a PS2 at 531.0 eV and a PS3 at 539.5eV and a PS4 at 540.3 eV and a PO1 at 532.7 eV. The PO1 band was mostly detected from rGO. The bands of PS1 and PS3 correspond to Sb 3d_5/2_ and PS2 and PS4 were observed by the interaction between Sb_2_S_3_ NRs and rGO. According to the PC2 of Sb_2_S_3_ NRs@rGO in Figure 4b and PS2 and PS4 of Sb_2_S_3_ NRs@rGO in Figure 4c, The PS2 and PS4 bands were shifted to higher binding energy because the C atoms in rGO have higher electronegative than Sb atoms in Sb_2_S_3_ NRs. The result of XPS was showed that Sb_2_S_3_ NRs are incorporated on rGO [23,24,25,26]. These results prove that Sb_2_S_3_ NRs@rGO was successfully synthesized. Raman spectra of GO and Sb_2_S_3_ NRs@rGO are presented in Figure 4d. The G and D bands of Sb_2_S_3_ NRs are clearly detected at 1586 cm^−1^ and 1330 cm^−1^, respectively, and a slight shift of the peaks of G and D band can be seen at 1594 cm^−1^ and 1334 cm^−1^, respectively, indicating the reduction of GO [27,28,29]. 

### 3.2. Sodium Storage Behavior

**Figure 5:**
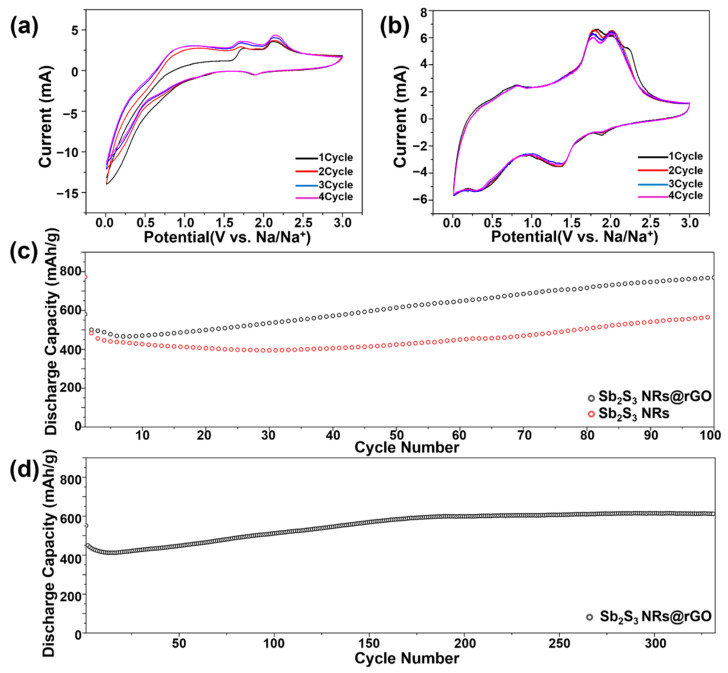
Cyclic voltammetry of (**a**) Sb_2_S_3_ NRs and (**b**) Sb_2_S_3_ NRs@rGO. Cycling performance of Sb_2_S_3_ NRs and Sb_2_S_3_ NRs@rGO at (**c**) 100D/100C and (**d**) 500D/500C.

The electrochemical performances of Sb_2_S_3_ NRs and Sb_2_S_3_ NRs@rGO as anode materials in SIBs were analyzed. Figure 5a shows the CV curve of Sb_2_S_3_ NRs over the first four cycles at a scanning rate of 0.05 mV/s between 0.01 and 3.0 V (versus Na/Na^+^). Compared with Figure 5a,b, the cathodic and anodic peaks of Sb_2_S_3_ NRs@rGO Figure 5b show remarkable intensity. As seen in the CV of Sb_2_S_3_ NRs Figure 5a, notable cathodic peaks were not observed at 0.3 V and 1.2 V but found in Sb_2_S_3_ NRs@rGO Figure 5b, which is attributed to the conversion reaction with sulfur (Sb_2_S_3_ + 6Na^+^ + 6e^−^ → 2Sb + 3Na_2_S) [29,30]. The anodic peaks centered at 1.1 V, 1.7 V, 2.1 V are related to the alloying reaction (2Na_3_Sb → 2Sb + 6Na^+^ + 6e^−^) and formation of Sb_2_S_3_ (2Sb + 3Na_2_S → Sb_2_S_3_ + 6Na^+^ + 6e^−^) in Figure 5a [29,30]. 

After the composite process with rGO, there is still the same change in the cathodic process, and more remarkable peaks around 0.7 V, 1.8 V, and 2.1 V were observed in Figure 5b [29,30]. The cycling performance of Sb_2_S_3_ NRs and Sb_2_S_3_ NRs@rGO were tested at 100 mAg^−1^ Figure 5c and 500 mAg^−1^ Figure 5d, respectively. Sb_2_S_3_ NRs deliver a discharge capacity of 564.42 mAh/g at 100 mAg^−1^ current density after 100 cycles. However, Sb_2_S_3_ NRs@rGO presents an improved cyclic performance of 769.05 mAhg^−1^ at the same conditions. As shown in Figure 5d, likewise at a higher current density of 500 mAg^−1^, the capacity can still attain 614.5 mAhg^−1^ after 300 cycles. From Figure 5d, the discharge capacity of Sb_2_S_3_ NRs@rGO decreases from the outset and increases gradually to 300 cycles, reaching the highest discharge capacity of 614.5 mAhg^−1^ at current of 500mAg^−1^. This value keeps maintaining after 300 cycles. The decrease of capacity could be explained by the formation of irreversible solid electrolyte interphase (SEI) layers from the decomposition of the electrolyte [31]. Due to its electronically insulating property, irreversible capacity fading could occur. The rise of capacity could be explained by several reasons. First of all, volume expansion of Sb_2_S_3_ induces the rise of the specific capacity. As shown in Figure S2a,d, SEM images of Sb_2_S_3_ NRs and Sb_2_S_3_ NRs@rGO were presented having homogeneous-width rod shapes. However, after several cycling tests, Sb_2_S_3_ NRs began to be expanded and defects and broken shapes were observed on its surface Figure S2b,c. As this expansion proceeds making some defects on the surface of Sb_2_S_3_ NRs, occluded reaction sites of Sb_2_S_3_ NRs also react with Na^+^. These mounting number of reaction sites induce the increase of the capacity [31]. Secondly, the activation of materials could also attribute to this phenomenon [32,33,34,35,36]. Besides, the SEI layer could be constructed to be more stable during the activation process above [37]. These factors contribute to the gradual rise of the specific capacity during repeated charge and discharge which is a common activation phenomenon for chalcogenide and oxide anodes [32,33,34,35,36]. Even though the rise of discharge capacity looks good, this structure instability causes the variableness of electric capacity for long cycling performances. As can be seen in Figure S2d,e, Sb_2_S_3_ NRs@rGO were also expanded, though the degree of swelling was quite smaller than that of Sb_2_S_3_ NRs and could keep its rod-like shapes after several cycle performances, suggesting that reduced graphene oxide (rGO) sustains over the volume expansion of Sb_2_S_3_ NRs during sodiation and desodiation. 

**Figure 6:**
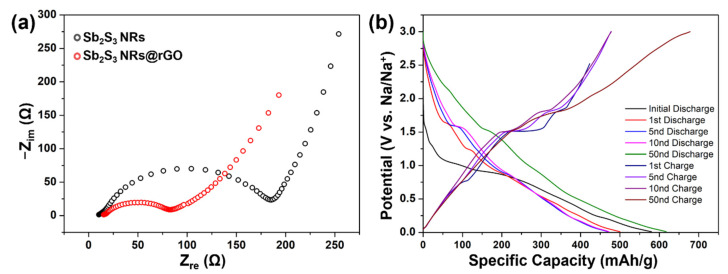
(**a**) EIS spectra of sodium ion batteries of Sb_2_S_3_ NRs and Sb_2_S_3_ NRs@rGO. (**b**) 1st, 5nd, 10nd, 50nd charge/discharge curve at a current density of 100 mAg^−1^ of Sb_2_S_3_ NRs@rGO.

To examine the electrochemical kinetics of the anode materials, the electrochemical impedance spectra (EIS) of Sb_2_S_3_ NRs and Sb_2_S_3_ NRs@rGO were tested, as shown in Figure 6a [38,39]. The ohmic resistance of Sb_2_S_3_ NRs@rGO is not more than that of Sb_2_S_3_ NRs, indicating a conductivity improvement. 

Figure 6b presents the galvanostatic charge/discharge profiles of Sb_2_S_3_ NRs@rGO at a current density of 100 mA/g, revealing the characteristic voltage profiles of the Sb_2_S_3_ NRs@rGO. As discussed above in Figure 5b, the conversion reaction with sulfur occurs at 0.3 V and 1.2 V, as presented by the two plateaus at similar voltages. Figure 6b alloying and formation of Sb_2_S_3_ occurs at 0.8 V, as shown by one plateau in Figure 6b. The initial discharge capacity is 580.4 mAh/g, while the second discharge delivers a capacity of 500.1 mAh/g, representing 86% coulombic efficiency. These different discharge capacities could be due to the solid electrolyte interface, which is irreversible. 

## 4. Conclusions

In summary, high gravimetric energy density and theoretical capacity (946 mA/g) of Sb_2_S_3_ has been introduced as high-performance anode material for sodium ion batteries. To overcome the volume expansion of the Sb_2_S_3_, 0.9 nm Sb_2_S_3_ NRs were synthesized and then made into a composite with reduced graphene oxide. Through this work, Sb_2_S_3_ NRs@rGO showed improved discharge capacity of 769.05 mAh/g at a current density of 100 mA/g after 100 cycles and excellent stability after 300 cycles, which was shown to be better than Sb_2_S_3_ NRs. This paper has developed a novel synthetic process for Sb_2_S_3_ NRs and Sb_2_S_3_ NRs@rGO and presents an avenue in determining more suitable anode materials for SIBs.

## Data Availability

The data presented in this study are contained within the article.

## Supplementary Materials

The following are available online at https://www.mdpi.com/article/10.3390/ma14247521/s1, Figure S1: XPS survey spectrum of Sb_2_S_3_ NRs and Sb_2_S_3_ NRs@rGO, Figure S2: SEM images of Sb_2_S_3_ NRs before cyclic test (a) and after several cyclic test (b), Table S1: EDS elemental qualification results, Table S2: Fine-scanned data of C 1s in rGO and Sb_2_S_3_ NRs@rGO, Table S3: Fine-scanned data of Sb 3d and O 1s in Sb_2_S_3_ NRs and Sb_2_S_3_ NRs@rGO.
