# Supplementary material for: Synthesis of Sb2S3 NRs@rGO Composite as High-Performance Anode Material for Sodium-Ion Batteries
Source: Materials (Basel). 2021 Dec 8;14(24):7521. doi: 10.3390/ma14247521 (PMC8707198; doi:10.3390/ma14247521)
Supplement: Supplementary file 1 [file materials-14-07521-s001.zip › materials-1480421-supplementary.pdf]

materials

MDPI

Article

Synthesis of Sb_2S_3 NRs@rGO Composite as High-Performance Anode Material for Sodium-Ion Batteries

Hosung Hwang [1], Honggyu Seong [1], So Yi Lee [1], Joon Ha Moon [1], Sung Kuk Kim [1], Jin Bae Lee [2], Yoon Myung [3], Chan Woong Na [3,*] and Jaewon Choi [1,*]

[1] Department of Chemistry and Research Institute of Natural Science, Gyeongsang National University, Jinju 52828, Korea; hshwang3@gnu.ac.kr (H.H.); gu9188@gnu.ac.kr (H.S.); oi_sioy115@gnu.ac.kr (S.Y.L.); answns36@gnu.ac.kr (J.H.M.); sungkukkim@gnu.ac.kr (S.K.K.)

[2] Korea Basic Science Institute, Daejeon 34133, Korea; jblee@kbsi.re.kr

[3] Dongnam Regional Division, Korea Institute of Industrial Technology, Busan 46744, Korea; myungyoon@kitech.re.kr

* Correspondence: cwna@kitech.re.kr (C.W.N.); cjw0910@gnu.ac.kr (J.C.)

Abstract: Sodium ion batteries (SIBs) have drawn interest as a lithium ion battery (LIB) alternative owing to their low price and low deposits. To commercialize SIBs similar to how LIBs already have been, it is necessary to develop improved anode materials that have high stability and capacity to operate over many and long cycles. This paper reports the development of homogeneous Sb_2S_3 nanorods (Sb_2S_3 NRs) on reduced graphene oxide (Sb_2S_3 NRs @rGO) as anode materials for SIBs. Based on this work, Sb_2S_3 NRs show a discharge capacity of 564.42 mAh/g at 100 mA/g current density after 100 cycles. In developing a composite with reduced graphene oxide, Sb_2S_3 NRs@rGO present better cycling performance with a discharge capacity of 769.05 mAh/g at the same condition. This achievement justifies the importance of developing Sb_2S_3 NRs and Sb_2S_3 NRs@rGO for SIBs.

Keywords: Sb_2S_3 NRs@rGO; anode materials; reduced graphene oxide; sodium ion batteries

The detailed morphology, structure and composition analysis

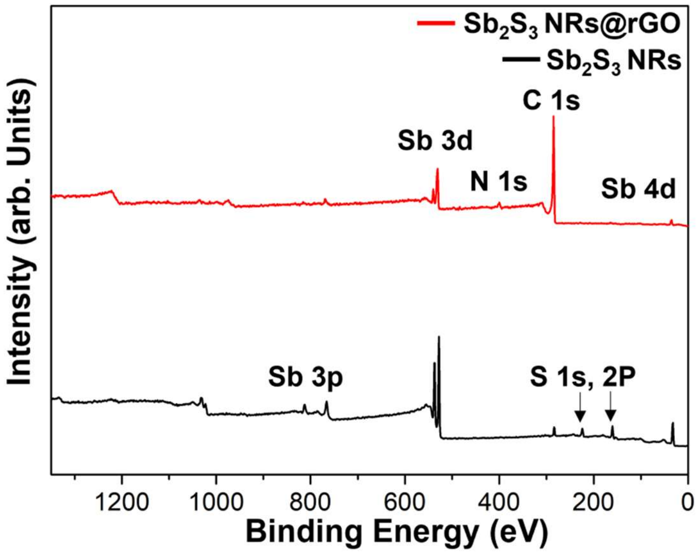

Figure S1. XPS survey spectrum of Sb_2S_3 NRs and Sb_2S_3 NRs@rGO.

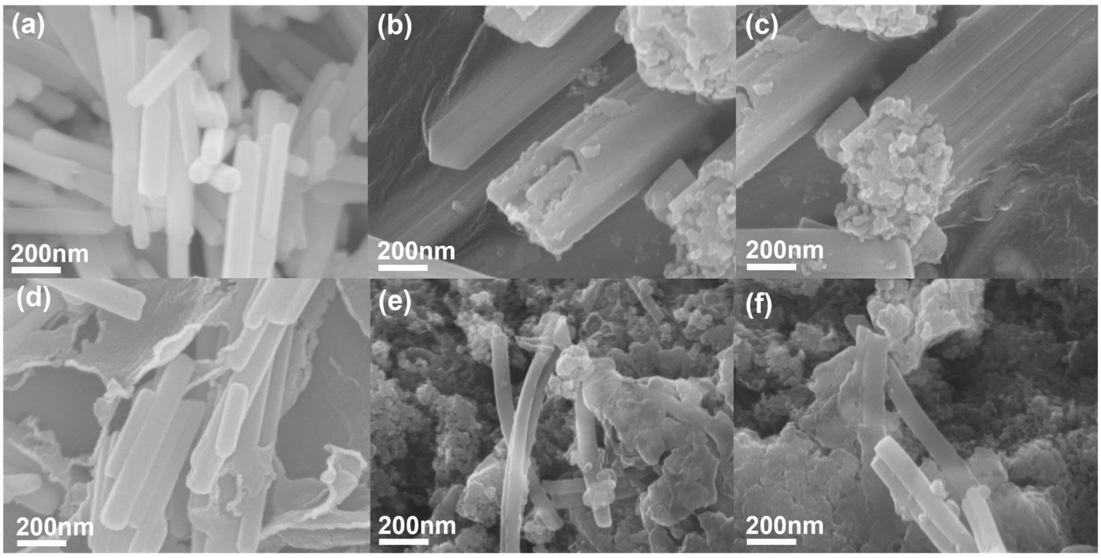

Figure S2. SEM images of Sb_2S_3 NRs before cyclic test (a) and after several cyclic tests (b), (c). SEM images of Sb_2S_3 NRs@rGO before cyclic test (d) and after several cyclic tests (e), (f).

Table S1. EDS elemental qualification results.

Element	Line Type	Apparent Concentration	k Ratio	Wt%	Wt% Sigma	Atomic %
**S**	**K series**	**0.22**	**0.00191**	**27.91**	**0.28**	**59.51**
**Sb**	**L series**	**0.48**	**0.00475**	**72.09**	**0.28**	**40.49**
**Total:**				**100.00**		**100.00**

Table S2. Fine-scanned data of C 1s in rGO and Sb_2S_3 NRs@rGO.

Sample	RGO		Sb2S3 NRs@rGO	
r^2	0.99930770		0.99887228	
Peak	PC1	PC2	PC1	PC2
Position (eV)	284.5	285.7	284.5	285.8
FWHM	1.173	1.258	1.201	2.093
Area (%)	86.7	13.3	75.1	24.9

Table S3. Fine-scanned data of Sb 3d and O 1s in Sb_2S_3 NRs and Sb_2S_3 NRs@rGO.

Sample	Sb2S3 NRs@rGO	Sb2S3 NRs@rGO				
r2	0.99804968	0.99804968				
Peak	PS1	PS1	PS1	PS1	PS1	PS1
Position (eV)	530.2	530.2	530.2	530.2	530.2	530.2
FWHM	1.670	1.670	1.670	1.670	1.670	1.670
Area (%)	28.0	28.0	28.0	28.0	28.0	28.0
